# A comparative gene analysis with rice identified orthologous group II *HKT* genes and their association with Na^+^ concentration in bread wheat

**DOI:** 10.1186/s12870-016-0714-7

**Published:** 2016-01-19

**Authors:** H. A. Chandima K. Ariyarathna, Klaus H. Oldach, Michael G. Francki

**Affiliations:** School of Plant Biology and Institute of Agriculture, The University of Western Australia, Crawley, 6009 Western Australia; State Agricultural Biotechnology Centre, Murdoch University, Murdoch, 6150 Western Australia; South Australia Research Development Institute, Plant Genomics Centre, Waite Research Precinct, Urrbrae, 5064 South Australia; Department of Agriculture and Food Western Australia, South Perth, 6151 Western Australia

**Keywords:** Group II *HKT*, IWGS, Rice genome, Na^+^ exclusion

## Abstract

**Background:**

Although the *HKT* transporter genes ascertain some of the key determinants of crop salt tolerance mechanisms, the diversity and functional role of group II *HKT* genes are not clearly understood in bread wheat. The advanced knowledge on rice *HKT* and whole genome sequence was, therefore, used in comparative gene analysis to identify orthologous wheat group II *HKT* genes and their role in trait variation under different saline environments.

**Results:**

The four group II *HKT*s in rice identified two orthologous gene families from bread wheat, including the known *TaHKT2*;*1* gene family and a new distinctly different gene family designated as *TaHKT2*;*2*. A single copy of *TaHKT2*;*2* was found on each homeologous chromosome arm 7AL, 7BL and 7DL and each gene was expressed in leaf blade, sheath and root tissues under non-stressed and at 200 mM salt stressed conditions. The proteins encoded by genes of the *TaHKT2*;*2* family revealed more than 93 % amino acid sequence identity but ≤52 % amino acid identity compared to the proteins encoded by *TaHKT2*;*1* family. Specifically, variations in known critical domains predicted functional differences between the two protein families. Similar to orthologous rice genes on chromosome 6L, *TaHKT2*;*1* and *TaHKT2*;*2* genes were located approximately 3 kb apart on wheat chromosomes 7AL, 7BL and 7DL, forming a static syntenic block in the two species. The chromosomal region on 7AL containing *TaHKT2*;*1 7AL*-*1* co-located with QTL for shoot Na^+^ concentration and yield in some saline environments.

**Conclusion:**

The differences in copy number, genes sequences and encoded proteins between *TaHKT2*;*2* homeologous genes and other group II *HKT* gene families within and across species likely reflect functional diversity for ion selectivity and transport in plants. Evidence indicated that neither *TaHKT2*;*2* nor *TaHKT2*;*1* were associated with primary root Na^+^ uptake but *TaHKT2*;*1* may be associated with trait variation for Na^+^ exclusion and yield in some but not all saline environments.

**Electronic supplementary material:**

The online version of this article (doi:10.1186/s12870-016-0714-7) contains supplementary material, which is available to authorized users.

## Background

Response to high saline conditions results from interaction of several biological processes controlled by multiple genes. Increasing evidence indicated that Na^+^ exclusion from the transpiration stream is an important mechanism associated with salt tolerance [1]. Na^+^ exclusion when measured as Na^+^ and/or K^+^ content in tissues or organs, is a robust and highly heritable trait in bread wheat [2]. The high affinity potassium transporter (*HKT*) genes are one of the most studied groups of membrane transporters in plants and the group I *HKT* genes that encode Na^+^ selective transporter proteins act in cohesion with the salt overly sensitive (SOS) pathway [3] identifying a major role in Na^+^ exclusion [2, 4] in wheat and other species [5–7]. Several group I *HKT* transporters are associated with retrieval of Na^+^ from xylem in root or sheath restricting transport and accumulation of salt in sensitive leaf tissues [1, 8, 9]. Grass species evolved a second class of HKT proteins encoded by group II *HKT* genes that function as Na^+^ and K^+^ permeable transporters [10]. A single member of this group, *TaHKT2*;*1*, has been identified from bread wheat that encoded a protein presumed to function in Na^+^ uptake from external medium through the roots [11, 12]. A recent study, however, showed that *TaHKT2*;*1* is a multigene family consisting of four functional genes and pseudogenes located on the long arm of homeologous group 7 chromosomes with evidence that the individual genes were not involved in controlling Na^+^ influx from the external medium into roots but may have a role in excluding Na^+^ from leaves or possibly involved in maintaining K^+^ status in the plant [13]. Therefore, a probable new capacity for group II *HKT* genes has been recognized where further research is warranted to gain additional insights into the role and potential of these genes in trait variation under different saline environments.

The most comprehensive analysis of group II *HKT* genes has been in rice (*Oryza sativa* L.) with up to four genes *OsHKT2*;*1*; *OsHKT2*;*2*, *OsHKT2*;*3*, and *OsHKT2*;*4* characterized for gene structure, expression and function. Some of the functional genes such as *OsHKT2*;*2* in *Indica* rice Pokkali [14] was identified as a chimeric gene, *No*-*OsHKT2*;*2*/*1*, in *japonica* rice Nona Bokra [15] and a truncated pseudogene in Nipponbare [14] that provided evidence of recent evolutionary changes in group II *HKT* genes in modern rice accessions. Phylogenetic relationships between rice group II *HKT* genes showed evidence of gene duplication and divergence, identifying two distinct clusters whereby the genes *OsHKT2*;*1* and functional *OsHKT2*;*2* diverged and clustered separately from *OsHKT2*;*3* and *OsHKT2*;*4* with >91 % DNA sequence identity within but only 40–50 % identity between clusters [5, 16–18]. Transcripts of the rice genes *OsHKT2*;*1* and *OsHKT2*;*2* were detected in roots with variable tissue expression in tolerant and susceptible rice varieties [19] whereas *OsHKT2*;*3* and *OsHKT2*;*4* transcripts accumulated in the shoot [17, 20]. Although *OsHKT2*;*1* is down regulated under saline conditions [21], there was no evidence to indicate a significant effect on the expression of the remaining rice group II *HKT* genes [17]. Most of the functional group II *HKT* genes in rice serve as Na^+^/K^+^ co-transporters with a role in maintaining K^+^/Na^+^ homeostasis in plants [20, 22, 23]. However, *OsHKT2*;*1* is an exception and presumed to function as a Na^+^ selective transporter with a putative role in “nutritional Na^+^ uptake” under K^+^ starvation [21]. The extensive knowledge on structure, expression and function of rice group II *HKT* genes, therefore, can be effectively used to identify and characterize gene orthologs in wheat based on comparative gene studies.

Phylogenetic relatedness of genes and whole genome sequence provides opportunities to identify gene orthologs across species. The advanced genomic resources available for rice including genome sequence data for 95 % of the 389 Mb genome with 37,544 annotated protein-coding genes [24] and integrated search tools that allow user-friendly access to genomic data enable a robust application of the rice genome in comparative gene studies with other cereal species. Although not as advanced as rice, the draft sequence of the 17Gb bread wheat genome identified >124,000 annotated and ordered gene loci [25] which has expedited comparative gene studies between rice and wheat to identify wheat genes and their association with biological processes controlling trait variation [26–30]. More specifically, the high degree of sequence conservation between *HKT* genes [10] allowed a comparative gene analysis within and across multigene families in the same [13], or different grass species [31]. Therefore, whole genome sequence from wheat and rice can be exploited in data mining and detailed comparative gene analysis for identification of wheat orthologs of the rice *HKT* genes.

While comparative gene analysis between rice and wheat enabled gene identification and characterization, determining function of wheat *HKT* orthologs defines their contribution towards improving salt tolerance. Quantitative trait loci (QTL) studies for salt tolerance in wheat [32–36] can be strategically utilized to investigate genes that may be functionally associated with trait variation. In particular, the doubled haploid (DH) mapping population derived from wheat cultivars Berkut and Krichauff as parents detected a number of QTL associated with physiological and yield related traits in controlled and field saline environments including 17 QTL for Na^+^ exclusion measured as leaf or shoot Na^+^ concentration in different environments [33, 37]. Interestingly, a member of the *TaHKT2*;*1* gene family was located in a similar region on chromosome 7AL [13] as QTL for shoot Na^+^ concentration and seedling biomass under controlled (hydroponics) saline conditions and in similar chromosomal regions for variation for yield components under moderate saline field environments [33, 37]. Therefore, QTL information can be used to make inferences on the role of wheat group II *HKT* gene orthologs in controlling phenotypes expressed under different saline environments, providing insights into their possible role in contributing towards improving salinity tolerance.

Although one multigene family *TaHKT2*;*1* was well characterized from wheat [13], given the fact that four individual genes exists in rice [17] it is reasonable to assume that wheat may have evolved multiple copies of more than one group II *HKT* gene family. The aim of this study, therefore, was to apply whole genome sequence in a comparative gene analysis to identify and characterize wheat orthologs of rice group II *HKT* genes. The association of wheat gene orthologs with trait variation was investigated by QTL analysis under different saline environments using the Berkut/Krichauff DH mapping population. The outcome of this study will increase our knowledge on the group II *HKT* genes in wheat and their significance on trait variation under different saline environments.

## Results

### Wheat genes orthologous to rice group II *HKTs*

Full length cDNA (FL-cDNA) of the rice genes *OsHKT2*;*1*, *OsHKT2*;*2*, *OsHKT*2;3 and *OsHKT*2;4 were used as query sequence in blastn and tblastx search of the IWGS survey sequence database in order to identify related wheat gene sequences. The four rice FL-cDNAs identified related wheat sequences on seven scaffolds (Table 1). The closely related FL-cDNA of *OsHKT2*;*1* and *OsHKT2*;*2*, both identified hits on six wheat scaffolds, three on wheat chromosome 7AL, two on 7BL and one on 7DL with up to 76 % DNA identity (Table 1) and in the same region as the known wheat group II HKT, *TaHKT2*;*1. TaHKT2*;*1* had 75–76 % DNA identity to both *OsHKT2*;*1* and *OsHKT2*;*2*. Similarly, FL-cDNA of *OsHKT2*;*3* and *OsHKT2*;*4* both had 80 % DNA sequence identity in the same region on three wheat scaffolds (Table 1). Therefore, a wheat group II *HKT* gene distinctly different to *TaHKT2*;*1* had identity with both *OsHKT2*;*3* and *OsHKT2*;*4*. The wheat scaffolds having sequence similarity with *OsHKT2*;*3* and *OsHKT2*;*4*, #4510252; #6569883 and #3312548, on chromosomes 7AL, 7BL and 7DL, respectively, were selected for further investigation.

**Table 1:** Wheat sequence scaffolds retrieved from IWGS having significant DNA identity (E = 0) with the FL-cDNA of rice group II *HKT* genes

Scaffolds number (chromosome arm)	Scaffold size (bp)	*OsHKT2*;*1* (Genbank #AB061311)	*OsHKT2*;*2* (Genbank #AB061313)	*OsHKT2*;*3* (Genbank #AJ491819)	*OsHKT2*;*4* (Genbank #AJ491854)
#4510252 (7AL)	10,610	3767–4922 (76 %)	3767–4922 (76 %)	8794–9915 (80 %)	8804–9915 (80 %)
#4434238 (7AL)	7049	3531–4667 (75 %)	3531–4667 (75 %)	*	*
#4523843 (7AL)	7561	4043–5179 (75 %)	4043–5179 (75 %)	*	*
#6657249 (7BL)	6544	2431–3586 (76 %)	2431–3586 (76 %)	*	*
#6748027 (7BL)	7204	3091–4246 (76 %)	3019–4246 (76 %)	*	*
#6569883 (7BL)	5298	*	*	1575–448 (80 %)	1565–448 (80 %)
#3312548 (7DL)	22,420	16,269–15,114 (76 %)	16,269–15,114 (76 %)	11,081–9960 (80 %)	11,071–9960 (80 %)

The region of similarity in the wheat scaffold is shown in bp and DNA sequence identity is in parenthesis. Scaffolds with no significant DNA sequence identity with rice FL–cDNA are shown by an asterix

**Fig. 1:**
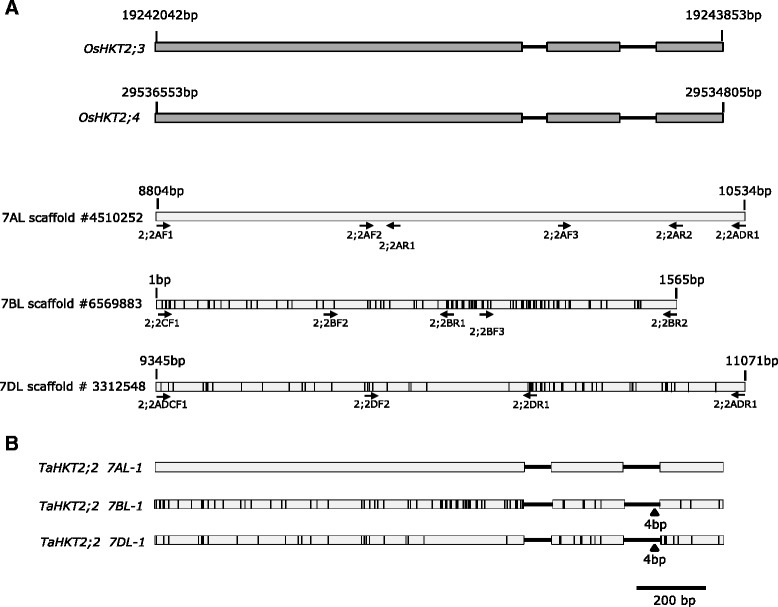
Wheat genes orthologous to rice *OsHKT2*;*3* and *OsHKT2*;*4*. **a** Gene structure of rice *OsHKT2*;*3* and *OsHKT2*;*4* showing 3 exons (*grey boxes*) interrupted by 2 introns (*black lines*). Wheat genomic sequence on scaffolds from 7AL, 7BL and 7DL with 80 % DNA identity to rice FL-cDNA of *OsHKT2*;*3* and *OsHKT2*;*4* are shown below the rice gene structures. The numbers at the top of each sequence represents the position of the gene on each scaffold. SNPs between wheat 7BL and 7DL relative to 7AL are indicated by black vertical lines. Position and direction of PCR primers to amplify wheat FL-cDNA are indicated by arrows. **b** Structure of the wheat genes, *TaHKT2*;*2 7AL*-1, *TaHKT2*;*2 7BL*-*1* and *TaHKT2*;*2 DL*-*1* deduced from the alignment of FL-cDNA sequences with cognate genomic scaffold sequences. Exons are shown in grey boxes and the introns in horizontal bars. SNPs are indicated by black vertical lines whereas black triangles represent INDELS relative to the gene on chromosome 7AL

The wheat sequences with identity to *OsHKT2*;*3* and *OsHKT2*;*4* on 7AL, 7BL and 7DL were analysed in multiple sequence alignments using the gene on 7AL as a reference to ascertain sequence variants that enabled design of gene specific reverse transcription polymerase chain reaction (RT-PCR) primer pairs (Table 2) to amplify FL-cDNA. The RT-PCR primers were strategically positioned against putative wheat exons assuming similar gene structure to *OsHKT2*;*3* and *OsHKT2*;*4* (Fig. 1a). Since the scaffold on 7BL did not appear to contain sequence corresponding to the full length sequence of *OsHKT2*;*3* or *OsHKT2*;*4*, the 3’-region of the gene on 7BL was amplified using primers designed from similar regions on 7AL and 7DL (Fig. 1a). Primer pairs showing sub-genome specificity were used to amplify partial but overlapping gene transcripts specifically from 7A, 7B and 7D and confirmed by nullisomic-tetrasomic (NT) analysis (data not shown). Subsequently, overlapping gene-specific cDNA from chromosome 7AL, 7BL and 7DL were amplified from root tissue, sequenced and assembled (Genbank accession numbers KR422354, KR422355 and KR422356 respectively). The FL-cDNA assembled for each gene on 7AL, 7BL and 7DL was aligned against the cognate genomic sequence from scaffolds #4510252; #6569883 and #3312548, respectively, to determine the intron-exon structure (Fig. 1b). The genes on chromosomes 7AL, 7BL and 7DL had similar structure to the *OsHKT2*;*3* and *OsHKT2*;*4* including 3 exons interrupted by 2 introns with intron splice sites having conserved motif GT and AG at the 5’ and 3’ boundaries, respectively. The cDNA of each gene had <64 % DNA sequence identity with *TaHKT2*;*1* gene family confirming that the two gene families were distinctly different. Therefore, the genes on 7AL, 7BL and 7DL were designated as *TaHKT2*;*2 7AL*-*1*, *TaHKT2*;*2 7BL*-*1* and *TaHKT2*;*2 7DL*-*1*, respectively. When compared with *TaHKT2*;*2 7AL*-*1*, all variants within exons were SNPs, totalling 69 for *TaHKT2*;*2 7BL*-*1* and 39 for *TaHKT2*;*2 7DL*-*1* whereas the comparison between *TaHKT2*;*2 7BL* and *TaHKT2*;*2 7DL* identified 76 SNPs.

**Table 2:** Gene specific primers and PCR annealing temperatures to amplify genomic and cDNA sequence from members of the *TaHKT2*;*2* gene family and qRT-PCR for *TaHKT2;1* and *TaHKT2;2* genes

	Forward primer			Reverse Primer	
Gene	Primer	Primer sequence (5’-3’)	Primer	Primer sequence (5’-3’)	Annealing Temperature °C
*TaHKT2*;*2 7AL*-*1*	2;2AF1	CCATCTATCTAACTCCAATGACTG	2;2AR1	CTCTCAGTGCCCATACCAT	54
*TaHKT2*;*2 7AL*-*1*	2;2AF2	TCAATACCATGCTCTTCACG	2;2AR2	AGACGCTCTGCTTCTCTGC	55–50
*TaHKT2*;*2 7AL*-*1*	2;2AF3	GGGGCAAACAAGAGAAGAA	2;2ADR1	CTAGCTCCTCTGCCTGTG	55–50
*TaHKT2*;*2 7BL*-*1*	2;2CF1	CTATCTAACTCCAATGCCTATC	2;2BR1	GCAAGTGGTTGAAACTCACAC	60–50
*TaHKT2*;*2 7BL*-*1*	2;2BF2	CTGTTGGCATACATGGTATC	2;2BR2	ATGGACAGTCCTACGTTCATA	65–55
*TaHKT2*;*2 7BL*-*1*	2;2BF3	CCGTTGCCATCACACTCTTG	2;2ADR1	CTAGCTCCTCTGCCTGTG	60–50
*TaHKT2*;*2 7DL*-*1*	2;2ADCF1	CACACTGCCATCTATCTAACTC	2;2DR1	ACAGTCTTCTCTTGTTCGCT	55–50
*TaHKT2*;*2 7DL*-*1*	2;2DF2	TGCTTTTCTCCATACCCA	2;2ADR1	CTAGCTCCTCTGCCTGTG	55–50
*TaHKT2*;*1 7AL*-*1*	2;1AF1	TCGGCTCTTATCAGAACACA	2;1AR1	CCACACGTTGATAGATAATGTC	55
*TaHKT2*;*1 7AL*-*1*	*qPCR2*;*1AF1*	ATGTCCCCTGCCATTGTAGAAT	*qPCR2*;*1AR1*	CGTGTTCTCATTGGTGGTTTTACTG	60
*TaHKT2*;*1 7AL*-*1*	*qPCR2*;*1BF1*	TGCGTTTTGCTAATTTGCCTG	*qPCR2*;*1BR1*	GATAAGAGCTGAGCCCATCCAAG	60
*TaHKT2*;*1 7DL*-*1*	*qPCR2*;*1DF1*	ACTGTTTTTCTCTCCTCAACGCTT	*qPCR2*;*1DR1*	TGCCTTTTGTGCTCGCTTC	60
*TaHKT2*;*2 7AL*-*1*	*qPCR2*;*2AF1*	GATATGGGCACTGAGAGGACTATGA	*qPCR2*;*2AR1*	AAACAGCATTTTATTCAGCGAGAT	60
*TaHKT2*;*2 7BL*-*1*	*qPCR2*;*2BF1*	GCTGCTTGAACTGGAATGCG	*qPCR2*;*2BR1*	GTGTTCTGTGATGCCCCTCTTGT	60
*TaHKT2*;*2 7DL*-*1*	*qPCR2*;*2DF1*	GATTCACTTGTCCTATTTTGTTGTCG	*qPCR2*;*2DR1*	GCAGGGAAACAAACATCTCTCTG	58
*TaActin*	*TaActin_qR*	TGGCACCCGAGGAGCACCCTG	*TaActin_qR*	GCGACGTACATGGCAGGAACA	60
*GAPDH*	*GAPDHF*	CGCCAGGGTTTTCCCAGTCACGAC	*GAPDHR*	TCAC ACAGGAAACAGCTATGAC	60
*TaEFA*	*TaEFA*_*qF*	GATTGGCAACGGCTACG	*TaEFA_qR*	CGGACAGCAAAACGACC	60

### Analysis of proteins encoded by *TaHKT2*;*2* genes

**Fig. 2:**
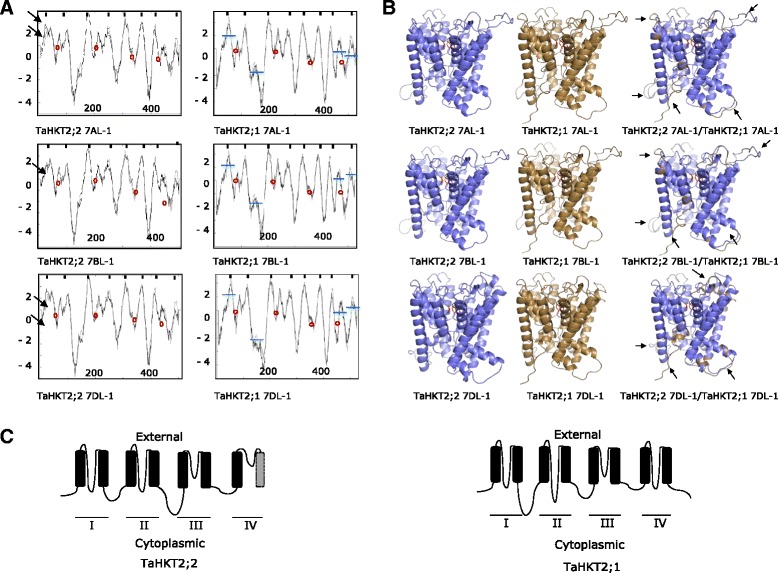
Predictions of transmembrane domains (TM) in proteins encoded by *TaHKT2*;*2* and *TaHKT2*;*1* genes. **a** Hydrophobicity plots of proteins encoded by each member of the *TaHKT2*;*2* and *TaHKT2*;*1* multigene families. The horizontal axis represents amino acid position and the vertical axis represents hydrophobicity value. TMs are indicated as black boxes on top of each graph. The region of the glycine filter domains are circled in red. Physical differences in the N-terminus shown by peak variation are indicated by black arrows. Differences in peak structures TaHKT2;1 relative to TaHKT2;2 proteins are indicated by horizontal blue lines. **b** The 3-D protein models of individual proteins encoded by *TaHKT2*;*1* and *TaHKT2*;*2* families and superimposed 3-D models of predicted proteins encoded by members of *TaHKT2*;*2* and *TaHKT2*;*1* from the same chromosome. Black arrows on the superimposed 3-D models represent different folding domains between proteins encoded by *TaHKT2*;*1* and *TaHKT2*;*2* counterparts on each chromosome. Conserved filter glycines are indicated in red. **c** Schematic diagram of the general model predicting protein topology deduced from proteins encoded by *TaHKT2*;*2* and *TaHKT2*;*1* genes. Black filled rectangles represent TMs and the lines cytoplasmic, external and P-loop domains. Putative TM (hydrophobicity value <0) is indicated by grey rectangle

**Fig. 3:**
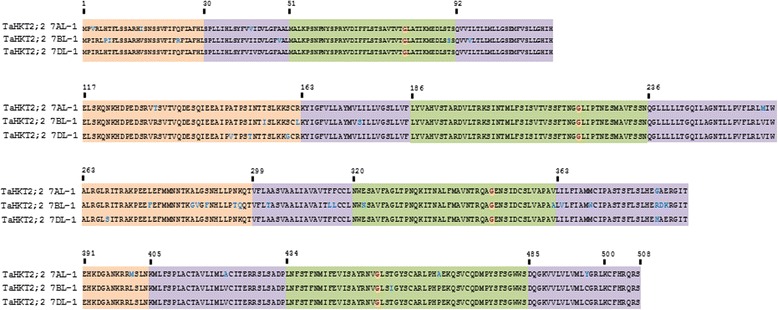
Multiple sequence alignment of predicted proteins encoded by *TaHKT2*;*2* genes from homeologous chromosome 7AL, 7BL and 7DL containing four membrane-pore-membrane structures (I to IV). Cytoplasmic, trans-membrane, and p-loop domains are in pink, purple and green backgrounds respectively. Conserved glycine residues representative of the cation selectivity filter are indicated in red. Differences in amino acid residues are shown in blue

Sequencing of FL-cDNA for *TaHKT2*;*2* enabled the predicted translation and subsequent characterization of encoded proteins. Each *TaHKT2*;*2* gene on chromosomes 7AL, 7BL and 7DL encoded a predicted protein of 508 amino acids. Combined analysis of hydrophobicity plots (Fig. 2a) and predicted 3D- structures (Fig. 2b) of the proteins encoded by *TaHKT2* genes allowed identification of protein folding patterns and modelling of protein topology. Superimposing models identified a number of differences in folding structure between each respective proteins encoded by *TaHKT2*;*1* and *TaHKT2*;*2* genes (Fig. 2b). The proteins encoded by each gene modelled a topology consisting of four sequentially arranged membrane-pour-membrane domains resembling core protein structure typical of HKT proteins (domains I–IV in Fig. 2c). The protein encoded by each *TaHKT2*;*2* gene was highly similar with 93–97 % amino acid identity and a high degree of sequence conservation in P-loops (Fig. 3). In each protein the conserved glycine molecules at Gly80, Gly221, Gly349 and Gly453 amino acid positions comprised the putative cation selectivity filter (Fig. 3). However, amino acid substitutions predicted variation in physical properties of the proteins including hydrophobic regions in the NH_2_-terminus encoded by *TaHKT2*;*2 7AL*-*1* and *TaHKT2*;*2 7DL*-*1* compared to *TaHKT2*;*2 7BL*-*1* (Figs. 2a and 3).

The proteins encoded by *TaHKT2*;*2* and *TaHKT2*;*1* were compared to ascertain similarities and differences. The proteins encoded by *TaHKT2*;*2* revealed less than 52 % amino acid identity with the proteins encoded by *TaHKT2*;*1* (Additional file 1: Figure S1), indicating that they are distinctly different. Although both protein families had a conserved structural core, protein topology and important functional domains, the protein encoded by *TaHKT2*;*2* relative to *TaHKT2*;*1* had truncated NH_2_-terminus, second cytoplasmic domain and carboxy-terminus, in addition to a four amino acid insertion in the third cytoplasmic domain (Additional file 1: Figure S1). Significant differences were identified in the carboxy-terminus whereby both hydrophobicity based analysis and in homology based 3D structures did not predict a transmembrane inner helix of 4th membrane-pore-membrane structure but a membrane embedded carboxy end in the proteins encoded by *TaHKT2*;*2* genes (Additional file 1: Figure S1). This is in contrast to the cytoplasm bound carboxy terminal in the proteins encoded by *TaHKT2*;*1*. In addition regions of amino acid variability in external and cytoplasmic caused distinct structural differences and these are depicted in Fig. 2c. Despite the different group II HKT proteins having conserved structural core and G-G-G-G type cation selectivity filter domains, variation in amino acid composition in the filter region (Additional file 1: Figure S1) and differences in cytoplasmic and external domains implied variability in functional characteristics.

### Quantitative expression of members of *TaHKT2*;*1* and *TaHKT2*;*2* multigene families

**Fig. 4:**
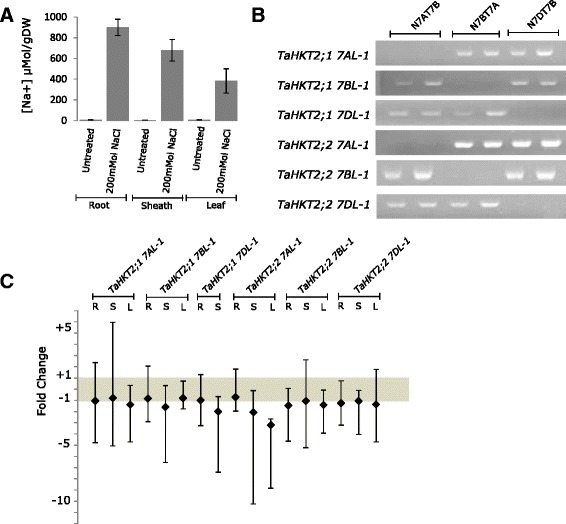
Expression of *TaHKT2*;*2* genes under non-treated and saline conditions. **a** Na^+^ concentration of tissue samples for qRT-PCR analysis. Na^+^ ion concentrations were measured in triplicate from four biological replicates for untreated (control) and 200 mM NaCl treated tissue samples. **b** Agarose gel electrophoresis showing sub-genome specificity of primer pairs used in qRT-PCR of group II *HKT* gene members using NT lines. Each line is in duplicate (**c**). Bar graph representing fold change estimated using the ∆∆C_T_ method. The error bars represent possible range of relative quantity values, RQ_max_ and RQ_min_, defined by the standard error of the ∆C_T_ **s** qRT-PCR for transcript analysis of group II *HKT* gene family members in wheat (Chinese Spring) using cDNA from root (R), sheath (S) and leaf (L) tissue samples untreated and treated for 72 h in 200 mM NaCl. The internal control genes *TaActin* was used to normalize for variability in initial RNA template for each reaction. The grey shaded region within the graph highlights the fold range that has no significant difference

*TaHKT2*;*1* and *TaHKT2*;*2* genes on chromosome 7AL, 7BL and 7DL were analysed for gene specific transcripts in roots, sheaths and leaf blade tissues of wheat seedlings (Chinese Spring). Significantly higher Na^+^ levels in leaf (cv = 3.26 %, *P* < 0.0001), root (cv = 3.35 %, *P* < 0.0003) and sheath (cv = 7.73 %, *P* < 0.0015) were observed for samples treated with 200 mM NaCl for 72 h compared with untreated control samples for each tissue (Fig. 4a) and, therefore, suitable for expression using quantitative RT-PCR (qRT-PCR). Sub-genome specific primer pairs (Table 2) were designed based on unique polymorphic sites and specificity was confirmed by NT analysis (Fig. 4b). The qRT-PCR analysis showed that members of the *TaHKT2*;*2* gene family and *TaHKT2*;*1 7AL*-*1* and *TaHKT2*;*1 7BL*-*1* were expressed in untreated root, sheath and leaf blade tissues and in the same tissue under NaCl stressed conditions (Fig. 4c). The exception, however, was *TaHKT2*;*1 7DL*-*1* where transcripts were detected in both sheath and root samples but not in the detectable limits of the qRT-PCR assay for the leaf blade in either the control or NaCl treated samples. Expression in control and 200 mM NaCl treated leaf blade (cv = 11.39 %, *P* > 0.88), root (cv = 13.75 %, *P* > 0.16) and sheath (cv = 15.97 %, *P* > 0.11) tissue samples showed that *TaActin* was suitable for gene expression normalization and was a reliable internal reference gene for quantification of *HKT* transcripts under salt stress. Fold change between control and NaCl treatments (∆∆C_T_ = -1 to +1) using *TaActin* as an internal reference showed no difference in expression of individual genes and it is likely that these genes were not salt responsive. However, a two-fold down regulation in *TaHKT2*;*1 7DL*-*1* expression and up to three-fold down regulation in *TaHKT2*;*2 7AL*-*1* expression levels indicated that these genes were differentially regulated in control and NaCl treated conditions (Fig. 4c).

### Physical mapping of wheat group II *HKT* genes and the promoter region of *TaHKT2*;*2* gene family

**Fig. 5:**
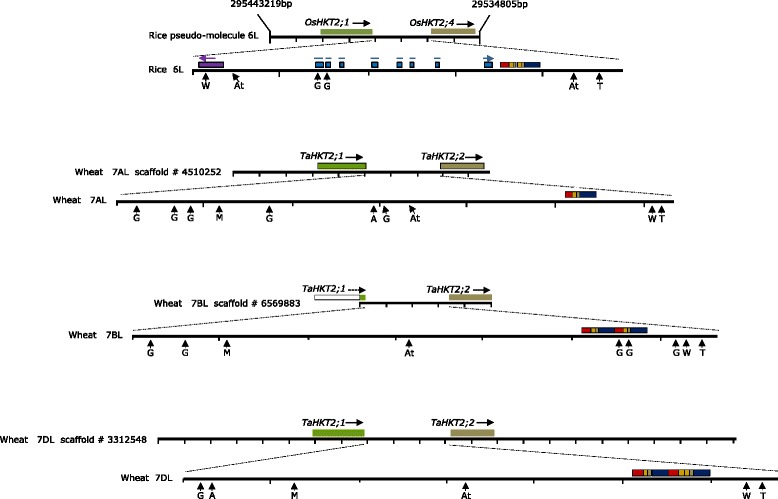
Comparative physical map of *OsHKT2*;*1* - *OsHKT2*;*4* intergenic region on rice chromosome 6L and *TaHKT2*;*1* - *TaHKT2*;*2* intergenic region on wheat chromosomes 7AL, 7BL and 7DL. Sequence is represented by chromosome region from pseudomolecule of rice chromosome 6L and scaffold sequences from wheat chromosomes 7AL, 7BL and 7DL where the scale intervals represents 1 kb. The direction of gene transcription is shown by black arrows. The intergenic region is expanded below each sequence or scaffold where scale intervals represent 500 base pairs. Tandem repeat units to the right of each expanded intergenic region are indicated by coloured boxes where each colour represents an individual repeat motif. PIF/ Harbinger type MITE elements are shown in purple whereas remnants of a CACTA type transposon “DTC_Isidor” are indicated in blue boxes. Salt induced *cis*-acting elements and other regulatory motifs identified on the *OsHKT2*;*4* and *TaHKT2*;*2* promoter regions are indicated by arrows, including ABRE (A), AtMYC2 (At), MYCATERD1 (M), GT-1 box (G), W-box (W) and TATA box (T)

Analysis of the pseudomolecule for rice chromosome 6L identified that *OsHKT2*;*1* (Ordered Locus Name, LOC_Os06g48810) and *OsHKT2*;*4* (LOC_Os06g48800) were separated by a physical distance of 2441 base pairs (Fig. 5). Similarly, *TaHKT2*;*1* and *TaHKT2*;*2* were identified on the same sequence scaffolds from chromosome 7AL (scaffold #4510252) and 7DL (scaffold #3312548) with intergenic distances of 3154 base pairs and 3330 base pairs, respectively (Fig. 5). *TaHKT2*;*2 7BL*-*1* and *TaHKT2*;*1* 7BL-1 genes were separated by an intergenic distance of 3302 base pairs, however, only partial sequence of *TaHKT2*;*1 7BL*-*1*, including the 3rd exon, 2nd intron and partial sequence of 2nd exon were identified on scaffold #6569883 (Fig. 5). Although a second copy of *TaHKT2*;*1 7BL*-*2* was retrieved from contig 3599841+ assembled in two different scaffolds (#6657249 and #16748027), neither of the scaffolds identified *TaHKT2*;*2 7BL*-*1*, hence, the wheat genome survey sequence did not contain a single scaffold with the entire length of both *TaHKT2*;*1* and *TaHKT2*;*2* genes on 7BL. Nevertheless, it was evident that *TaHKT2*;*1* and *TaHKT2*;*2* genes in wheat are physically linked on 7AL, 7BL and 7DL with intergenic distances, comparable to *OsHKT2*;*1* and *OsHKT2*;*4* genes on rice chromosome 6L.

The similar size of the intergenic regions between the rice genes *OsHKT2*;*1* and *OsHKT2*;*4* and wheat genes *TaHKT2*;*1* and *TaHKT2*;*2* prompted a further comparison of the nucleotide sequences. The intergenic region on 7AL, 7BL and 7DL had <40.8 % DNA identity to the intergenic region between *OsHKT2*;*1* and *OsHKT2*;*4* in rice, indicating significant diversity in this region. A blastn and tblastx analysis in NCBI did not identify sequence similarity with any expressed genes in the intergenic regions for rice or wheat. However, a self-by-self blastn search identified direct, tandem imperfect repeats in the intergenic region (approximately 400 base pairs upstream of the translation start site of *TaHKT2*;*2*) of rice *OsHKT2*;*4* and wheat *TaHKT2*;*2* -*7BL* and *TaHKT2*;*2* -*7DL* where each repeat motif was less than 70 base pairs in size (Fig. 5, Additional file 2: Figure S2). However, only one copy of two imperfect motifs was represented in the intergenic region on 7AL (Fig. 5, Additional file 2: Figure S2). Furthermore, a complete PIF/Harbinger type miniature inverted transposon element (MITE), *DTH*_*Ors48*, was identified in the 5’ region of the rice intergenic sequence and remnants of a *DTC*_*Isidor* type DNA transposon (TREP accession number TREP3425) in the central region (Fig. 5, Additional file 2: Figure S2) but no transposon related elements were identified in the intergenic region of wheat 7AL, 7BL or 7DL.

The *TaHKT2*;*1* and *TaHKT2*;*2* intergenic region also represented the promoter region for *TaHKT2*;*2* genes on 7AL, 7BL and 7DL and enabled comparative analysis of putative stress regulatory elements for genes on homoeologous chromosomes. The wheat *TaHKT2*;*2* promoter region revealed 67–88 % sequence identity between promoters of genes on homoeologous chromosomes (Additional file 2: Figure S2). Conserved *cis*-acting regulatory elements (CRE) were identified including three major salt induced CREs, W-box, GT-box and AtMYC2 in the promoter regions on wheat chromosomes 7AL, 7BL and 7DL with similar elements also represented in the promoter region of rice *OsHKT2*;*4* (Fig. 5, Additional file 2: Figure S2). Furthermore, the *TaHKT2*;*2* promoter identified additional CRE including MYCATRED1 and ABRE on 7AL, 7BL and 7DL but were not evident in the promoter region of rice (Fig. 5, Additional file 2: Figure S2). Therefore, the CRE elements indicated a role for salt- activated transcription factors (TF) in regulation of *TaHKT2*;*2* genes and orthologs in rice but with variable frequency of the individual elements.

### Functional Association of wheat group II *HKT* genes

**Fig. 6:**
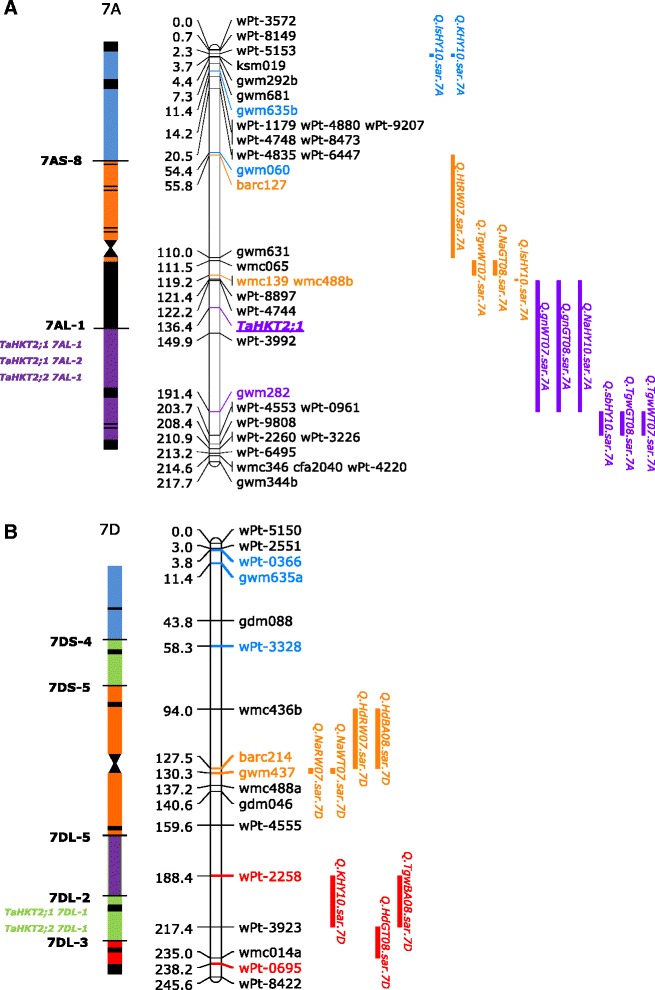
Deletion bin and genetic maps of chromosome 7A (**a**) and 7D (**b**) and allocation of *TaHKT2*;*1* and *TaHKT2*;*2* genes and QTL in similar regions under different saline environments. Allocation of *TaHKT2*;*1* and *TaHKT2*;*2* in the deletion bin map for each chromosome is presented on the left whereas the chromosomal linkage map with position of *TaHKT2;1* (bold and underlined) and the QTL positions derived from Berkut/Krichauff DH mapping population [33, 37] is represented on the right. *TaHKT2*;*1* and *TaHKT2*;*2* genes, QTLs and flanking markers are colour coded according to their allocation within deletion bins [79, 80]. QTL detected for phenotypes in different saline environments include shoot Na^+^ concentration (*Q.Na*), thousand grain weight (*Q.Tgw*), seedling biomass (*Q.sb*), grain number per m^2^ (*Q.gn*), leaf symptoms (*Q.ls*), plant height (*Q.Ht*), Heading date (*Q.Hd*) and shoot K^+^ concentration (*Q.K*) as previously reported [37]

An association of *TaHKT2*;*2* genes with trait variation under salt stress was investigated using deletion and genetic mapping. Deletion line analysis showed that *TaHKT2*;*2* genes were assigned to the distal end of chromosomes 7AL and 7DL and the proximal region of 7BL (data not shown) and the same chromosome bins as *TaHKT2*;*1* [13]. Therefore, functional analysis based on deletion line analysis previously reported [13] indicated that *TaHKT2*;*2* is associated with similar phenotypes as *TaHKT2*;*1* under salt stress. In order to further discriminate whether *TaHKT2*;*1* and *TaHKT2*;*2* were associated with specific trait variation under salt stress in different environments, QTL detected in response to low and high salt stress in Berkut/Krichauff DH mapping population [33, 37] were integrated onto the deletion map based on the chromosomal bin location of flanking markers. A total of six QTL were aligned to the distal bin on chromosome 7AL and in the same region as *TaHKT2*;*1 7AL*-*1* and *TaHKT2*;*2 7AL*-*1*, however, neither *TaHKT2*;*1* nor *TaHKT2*;*2* genes were co-located with QTL in the same chromosomal bin on 7DL (Fig. 6) and QTL were not identified on 7BL. Therefore, the co-location of genes and QTL in the same chromosomal region indicated that *TaHKT2*;*1 7AL*-*1* and *TaHKT2*;*2 7AL*-*1* may contribute to variation for leaf/shoot Na^+^ concentration, 1000 grain weight, grain number per m^2^ and seedling biomass on 7AL in response to specific saline environments.

**Fig. 7:**
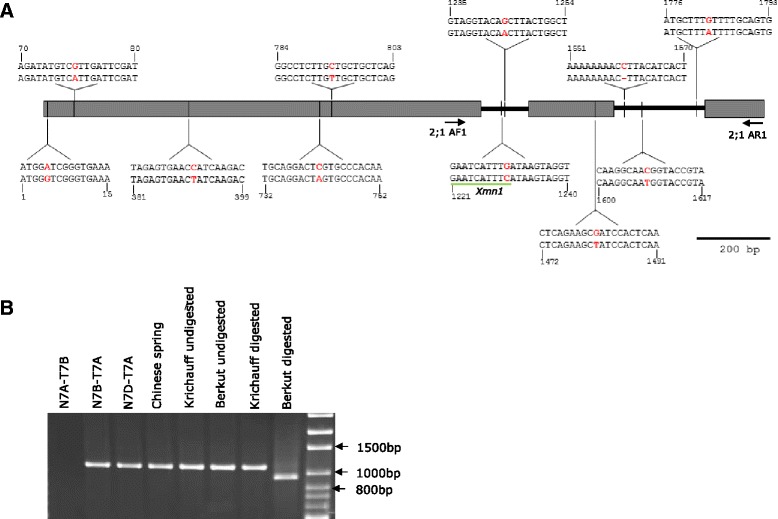
DNA sequence variability in the *TaHKT2*;*1 7AL*-*1* gene between wheat cultivars Krichauff and Berkut. **a** Intron-exon structure where exons are represented as grey boxes and introns represented by black lines. SNPs are indicated by black vertical lines. DNA sequence and position (in base pairs) flanking each SNP is shown, with top sequence representing Krichauff and bottom sequence representing Berkut. SNP variation within the restriction enzyme recognition site, *Xmn*1, is underlined. Location of gene specific PCR primer pair for the *TaHKT2*;*1 7AL*-*1*, (2;1 AF1 and 2;1 AR1) to amplify the SNP at 1230 base pairs is shown by black arrows. **b** Agarose gel electrophoresis of *TaHKT2*;*1 7AL*-*1* gene specific CAPS marker showing specificity to chromosome 7A using NT analysis and size difference of amplicons for Berkut and Krichauff parents following digestion with *Xmn*I. The DNA ladder is shown to the right of the figure

The association of *TaHKT2*;*1 7AL*-*1* and *TaHKT2*;*2 7AL*-*1* with specific trait variation was further investigated using genetic mapping analysis. *TaHKT2*;*1 7AL*-*1* and *TaHKT2*;*2 7AL*-*1* were PCR amplified and sequenced from Berkut and Krichauff to identify polymorphism and to develop gene specific markers for mapping in the DH population. Single nucleotide polymorphism (SNP) or insertion-deletion (INDEL) polymorphisms between Berkut and Krichauff were not identified in exons or introns for *TaHKT2*;*2 7AL*-*1*, indicating that no protein differences encoded by this gene were associated with trait variation on chromosome 7AL and, therefore, was excluded from further analysis in the DH population. However, *TaHKT2*;*1 7AL*-*1* identified 11 SNPs between Berkut (Genbank accession number KR422357) and Krichauff (Genbank accession number KR422358) (Fig. 7a). A SNP at 1230 base pairs from translation start site identified a restriction site for *Xmn1* enzyme in Berkut but not in Krichauff and this was used to develop a marker for *TaHKT2*;*1 7AL*-*1* (Fig. 7a). A cleaved amplified polymorphic sequence (CAPS) marker was developed using a 3’ mismatch PCR primer pair, 2;1AF1 and 2;1AR1 (Table 2), designed to amplify a 1103 base pairs fragment containing the SNP at 1230 base pairs The sub-genome specificity of the PCR fragment was confirmed by NT analysis (Fig. 7b). The *TaHKT2*;*1 7AL*-*1* specific CAPS marker identified a 922 base pairs DNA fragment for Berkut and 1103 base pairs DNA fragment for Krichauff parents following digestion with *Xmn*1 (Fig. 7b). The Berkut/Krichauff mapping population (150 DH lines) was genotyped for the *TaHKT2*;*1 7AL*-*1* specific CAPS marker and the data was integrated into existing genetic map containing 34 markers on chromosome 7A. *TaHKT2*;*1 7AL*-*1* mapped on chromosome 7AL at a genetic distance of 136.4 cM and flanked by markers *wpt*-*4744* (122.2 cM) and *wpt*-*3992* (149.9 cM) and within the QTL intervals for shoot Na^+^ concentration (*Q.NaHY10.sar.7A*) and grain number per m^2^ (*Q.gnWT07.sar.7A*, *Q.gnGT08.sar.7A*) detected under different saline environments (Fig. 6a). Therefore, based on genetic mapping and QTL analysis, it is reasonable to assume that *TaHKT2*;*1 7AL*-*1* was associated with specific trait variation but not under all saline environments.

**Fig. 8:**
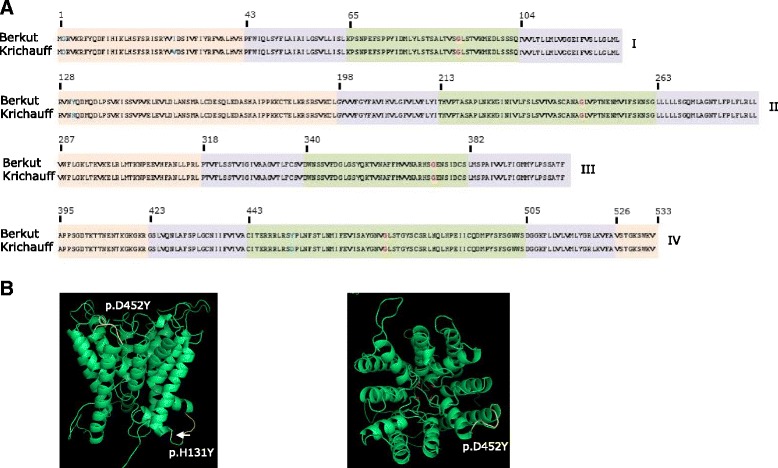
Predicted proteins encoded by *TaHKT2*;*1 7AL*-*1* in cultivars Krichauff and Berkut. **a** The cytoplasmic, trans-membrane, and p-loop domains are identified in pink, purple and green backgrounds respectively. Conserved glycine residues representative of the cation selectivity filter are indicated in red. Amino acid differences at residues 2, 27, 131 and 452 are in blue. **b** Predicted 3D models of superimposed proteins encoded by *TaHKT2*;*1* from Krichauff and Berkut, side view (*right*) and top view (*left*). The yellow regions indicate differences in the p-loop (p.D452Y) and cytoplasmic (p.H131Y) domains between Berkut and Krichauff and the position of cation selectivity filter glycine molecules are indicated in red

Since *TaHKT2*;*2 7AL*-*1* did not show any differences in amino acid residues, the predicted proteins encoded by Berkut and Krichauff focused on *TaHKT2*;*1 7AL*-*1* alleles and were further analysed to identify protein folding differences that may be related to trait variation. The encoded proteins were predicted from gene sequences derived from each parental allele and using FL-cDNA from Chinese Spring as a reference. Four amino acid substitutions between Berkut and Krichauff were identified at amino acid residues 2, 27, 131 and 452 (Fig. 8a). The 3-D protein modelled by superimposing amino acid sequences from Berkut and Krichauff predicted differences in cytoplasmic (p.H131Y) and the p-loop (p.D453Y) domains (Fig. 8b) that anticipated functional differences between the proteins.

## Discussion

Rice contains four individual group II *HKT* genes and it was expected that a total of at least 12 orthologs would be identified in wheat assuming one copy of each ortholog on the A, B and D genomes, with relatively similar sequences but not necessarily identical function. Instead, however, a genome-wide comparative gene analysis in this study identified only two distinct group II *HKT* gene families in wheat including the previously described *TaHKT2*;*1* family [13] and a new gene family, *TaHKT2*;*2*, that is distinctly different from *TaHKT2*;*1*. Therefore, it appears that wheat and rice group II *HKT* genes evolved by gene duplication but diverged independently possibly reflecting their involvement in different biological processes across species.

The common structure in barley, rice and Arabidopsis [10] makes it reasonable to hypothesise that the ancestral *HKT* gene of plants consisted of three exons. The comparative gene analysis in wheat and rice described in this study supports this hypothesis and the ancestral gene subsequently duplicated and diverged independently in the two species. This study revealed that the two rice group II *HKT* gene clusters, with each comprising of two genes based on phylogenetic relationships [5, 16, 17], have orthologous gene families in wheat. The wheat *TaHKT2*;*2* gene family had only one copy on each sub-genome and did not show evidence of gene duplication prior to or after polyploidization. However, duplication with subsequent translocation and divergence was evident for the rice ortholog *OsHKT2*;*3* on pseudomolecule 3 and *OsHKT2*;*4* on pseudomolecule 6. On the other hand, evidence indicated tandem gene duplication in A and B progenitor genomes predated polyploidization resulting in multiple copies of *TaHKT2*;*1* gene family on wheat chromosome arms 7AL and 7BL including divergence and pseudogenization [13] similar to the orthologous rice genes *OsHKT2*;*1* and *OsHKT2*;*2* on chromosome 6. Therefore, it is apparent that gene duplication of a common ancestral *HKT* gene was a key mechanism contributing in the creation of group II *HKT*, however, gene copies were possibly subjected to different selective constraints whereby divergence did not evolve in parallel during domestication of modern wheat and rice.

Analysis of the intergenic regions between *TaHKT2*;*2* and *TaHKT2*;*1* and the orthologous rice genes *OsHKT2*;*1* and *OsHKT2*;*4* provided further insights on evolution of group II *HKT* genes across species. Three typical structural organization categories in grass genomes have been identified based on analysis of DNA content within large genomic fragments between genes [38]. The physical size of intergenic regions can vary significantly between conserved coding regions in two or more grass species, ranging from a few to a tens of kilobases in size largely due to differential accumulation of transposable and other repetitive elements [39]. Alternatively, regions of the genome can have high gene content whereby coding regions and the size of the physical distance between genes are conserved [40], indicating that genome regions may not necessarily be prone to proliferation of transposable and other repetitive elements. Finally, there are both conserved high gene density with similar intergenic regions across species interspersed with large intergenic regions varying in size [41]. This study identified that the group II *HKT* genes in wheat and rice have similar physical distance in the intergenic region and, therefore, evolutionary mechanisms are proposed to maintain genes physically close together across species possibly as a means to preserve gene regulation, co-expression or co-functionality [42, 43]. A MITE and remnants of a transposable element was identified in the intergenic region between *OsHKT2*;*1* and *OsHKT2*;*4* in rice indicating that retrotransposition events may have been evident but were not sustained and, consequently, eliminated during evolution leaving a transposon “footprint”. Remnants of transposable elements were not evident in the orthologous region in wheat but instead, low complexity imperfect repeats were the major features in the intergenic region between wheat *TaHKT2*;*1* and *TaHKT2*;*2* in wheat 7BL, 7DL and in rice but absent in wheat 7AL. The differences observed in short repeat motifs between chromosomes and species can arise from replication misalignment and polymerase slippage resulting in deletion or addition of repeat units [44, 45]. It is, therefore, evident that the intergenic region between *TaHKT2*;*1* and *TaHKT2*;*2* in wheat and the orthologous region between *OsHKT2*;*1* and *OsHKT2*;*4* in rice is not conducive to major changes in size and conservation of physical distance within and across species may be implicated in the preservation of some gene functions.

This study provided evidence to support the hypothesis that the similar physical closeness of genes in rice and wheat is an evolutionary means to maintain similar gene regulation across species. *TaHKT2*;*2* gene family was expressed in roots, sheath and leaf blade tissues whereas *TaHKT2*;*1* genes were expressed in roots and sheath but with a low transcript abundance in leaf blade. Orthologous rice genes showed similar expression patterns where *OsHKT2*;*4* was expressed in root, sheath and leaf blade tissues [20] and *OsHKT2*;*1* showed expression in roots and sheath but down regulated in leaf blades [21], despite variability in the number and types of CRE in the intergenic regions between species. Therefore, it is plausible that similar small physical distance between orthologous *HKT* genes could be an evolutionary mechanism to conserve gene expression in rice and wheat. Interestingly, the consistent expression and maintenance of transcript levels indicated that the two wheat group II *HKT* gene families may interact in coordinated manner and possibly in cohesion with other genes such as group I *HKT* genes regardless of saline environments. Similar gene interactions resulting in an organized response to salt stress has been observed for rice *HKT* genes *OsHKT1*;*4* and *OsHKT1*;*5* [46] providing a transcript-coordinated model for wheat *HKT* genes. The manner by which group II *HKT* genes are coordinated in a biological system in response to salinity is yet to be realized and warrants further investigation.

The intergenic region between the two group II *HKT* genes on each homeologous chromosome also occupy the promoter region for *TaHKT2*;*2* genes. In addition to the variable number of CRE present in different homeologs, specific direct imperfect repeat motifs within the promoter region can act as suppressors or enhancers of gene transcription often by functioning as putative transcription factor-binding domains [47–50]. Interestingly, tandem imperfect direct repeats important for gene expression and regulation were identified in the promoter of the *HKT* gene from *Arabidopsis thaliana*, *AtHKT1*;*1*, whereby variation or absence of the repeats resulted in significant transcriptional response to elevated NaCl [51]. Therefore, further studies on the short repetitive elements is warranted to determine their role in regulation of *TaHKT2*;*2* transcription during different stages of plant development beyond that analysed in this study and under different saline conditions.

The fold difference in transcript abundance for *TaHKT2*;*1 7DL*-*1* and TaHKT2;2 7AL-1 may indicate their involvement in controlling Na^+^ accumulation in a tissue specific manner. Despite variable and multiple presence of salt induced CREs in promoter regions, the majority of group II *HKT* genes did not show a significant change in transcription in roots, sheath and leaf blades in response to NaCl treatment under the experimental conditions in this study. The lack of response to salt, therefore, raises some doubt as to the transcriptional significance of some *TaHKT2* genes in Na^+^ transport in roots, sheath or leaf under hydroponic conditions. A similar outcome was observed for the group I *HKT* member where *TmHKT1*;*4* did not show variability in transcript abundance under saline conditions but was co-located with a QTL for Na^+^ exclusion accounting for 25 % of the phenotypic variation indicating that transcript abundance may not solely explain gene action in controlling Na^+^ levels [52]. We cannot exclude the possibility that some group II *HKT*s are regulated under salt stress in other tissues and different environments or may, indeed, play a non-transcriptional role in controlling Na^+^ accumulation. Therefore, systematic and integrated studies involving whole plant analysis would provide a more comprehensive understanding of the co-ordinated transcriptional regulation and interactions of all members of the group II *TaHKT* gene family that control Na^+^ accumulation.

Distinct differences between the proteins encoded by two group II *HKT* gene families in wheat predicted variation in their function. The critical functional domains of the protein encoded by *TaHKT2*;*1 7BL*-*1* was shown to be important for cation selectivity and ion transport properties [53–56] and used as a basis to infer functional variation in proteins encoded by *TaHKT2*;*2*. Structural modelling and superimposing the known functional domains responsible for Na^+^ and K^+^ transport in the protein encoded by *TaHKT2*;*1 7BL*-*1* identified variants in proteins encoded by *TaHKT2*;*2*. The NH_2_ and carboxy-termini are essential functional domains in HKT proteins affecting K^+^ selectivity [54]. The length and amino acid composition of the NH_2_ and carboxy termini were distinctly different in the two protein families and it is possible that the truncated domains could result in either non-functional proteins or have significantly modified function. Highly conserved, positively charged amino acids in the inner helix of the 4th TM-P-TM domain are critical for formation of the salt bridge and K^+^ selective transporter activity [57, 58]. However, HKT-related proteins between species have similar structural core and these positively charged amino acids are not present in plant K^+^ channels [57]. Therefore, further studies such as heterologous expression would be worthwhile to decipher whether the truncated carboxy termini in proteins encoded by *TaHKT2*;*2* have transporter activity with channel-like properties. Interestingly, protein encoded by the rice ortholog *OsHKT2*;*4* exhibited variable kinetics in heterologous expression, including a calcium-permeable weak selective cation channel that provided evidence for alternative functional modes for group II HKT proteins [20, 22, 59]. Moreover, differences in the cytoplasmic and external domains arising from single amino acid substitutions between allelic variants were identified in the protein encoded by rice gene *OsHKT1*;*4* [60] and *Arabidopsis AtHKT1*;*1* [61] and similar variations predicted functional differences within the protein family encoded by *TaHKT2*;*2* compared with proteins encoded by *TaHKT2*;*1*. In addition to a number of amino acid substitutions in the cytoplasmic and external domains, the protein encoded by *TaHKT2*;*2* identified an alanine substitution for serine at amino acid residue 216 compared to the protein encoded by *TaHKT2*;*1 7BL*-*1* that can cause significant effects on ion transport activity. The substitution of Ala_216_ near entrance of the pore region reduced Na^+^ transport capacity the protein encoded by *TaHKT2*;*1 7BL*-*1* [62] and, therefore, a similar change at this residue implies a modified ion transport activity of proteins encoded by *TaHKT2*;*2*. Therefore, whilst the conserved protein structural core and the shared G-G-G-G type cation selectivity filter predicted some functional similarity between *TaHKT2*;*2* and *TaHKT2*;*1* (for example, ability to transport both K^+^ and Na^+^), the encoded proteins may have diversity for ion selectivity based on modified protein domains and amino acid changes in critical functional domains.

In-planta function of group II *HKT* transporters is poorly understood in wheat but phenotypic analysis of aenuploid lines under salt conditions provided new knowledge on their potential role in controlling ion accumulation in the plant. Wheat deletion lines null for *TaHKT2*;*1* on chromosomes 7AL, 7BL and 7DL did not show significant difference in root Na^+^ concentration and, therefore, members of this gene family were proposed to be involved in Na^+^ transport from root to sheath and regulation of K^+^ in different tissues rather than import of ions from the external medium through the roots [13]. Since members of the *TaHKT2*;*2* gene family were allocated to the same deletion bin as *TaHKT2*;*1* on chromosomes 7AL, 7BL and 7DL, it was apparent that neither *TaHKT2*;*2* nor *TaHKT2*;*1* genes were responsible for primary root Na^+^ uptake from an external medium despite both genes being expressed in roots. However, either or both genes could be involved in regulating Na^+^ from root to sheath or maintaining K^+^ levels under untreated and salt treated conditions. Interestingly, the *TaHKT2*;*2* ortholog in rice, *OsHKT2*;*4* mediated a robust low affinity K^+^ transport in a heterologous expression system [59] yet *OsHKT2*;*4* knockout mutants did not confer a similar phenotype under different ionic conditions [22], indicating that this gene is capable but not critical for transporting K^+^ in the plant and more likely reliant on the contribution of other genes and proteins in a more complex system. The functional association of *TaHKT2*;*1* and *TaHKT2*;*1* with trait variation, therefore, was further investigated.

Deletion bin and genetic mapping coupled with QTL analysis was used as a guide to associate group II *HKT* genes with trait variation under saline environments. Although these resources provided low resolution analysis, we were able demonstrate that *TaHKT2*;*1* and *TaHKT2*;*2* genes were not associated with some traits under saline environments on chromosomes 7BL and 7DL. However, the location of *TaHKT2*;*2* and *TaHKT2*;*1* on 7AL were identified in the same chromosomal deletion bin as QTL for leaf/shoot Na^+^ concentration and yield measurements under different saline environments [33, 37], indicating their potential involvement in phenotype variation. Exons and introns in *TaHKT2*;*2 7AL*-*1* were identical between Berkut and Krichauff and, consequently, were unable to be assigned to the genetic map in the DH population. It would, however, be premature to conclude that *TaHKT2*;*2 7AL*-*1* does not affect specific phenotypes as the gene may have transcriptional, post-transcriptional or post-translational regulation during different stages of plant development that could affect phenotypic variation under different saline environments. DNA sequence variability within introns and exons of *TaHKT2*;*1 7AL*-*1* allowed a low resolution but genetic association with QTL, predicting a role for this gene in controlling Na^+^ exclusion under hydroponic and field conditions, grain number and grain weight in specific saline environments. However, the QTL has a large genetic and physical distance and we cannot exclude the possibility that genes other than *TaHKT2*;*1 7AL*-*1* in the interval contribute to phenotypic variation. Fine resolution genetic mapping would confirm the role of *TaHKT2*;*1 7AL*-*1*and other genes in controlling variation for some traits under different saline environments. Although a further three QTL were detected within the same deletion bin, *TaHKT2*;*1 7AL*-*1* did not co-locate within the same mapping interval and, therefore, unlikely to be associated with phenotypic variation for thousand grain weight and seedling biomass under different saline conditions. The genetic control of these traits, therefore, was likely caused by genes other than the *TaHKT2* family. A structural model and variability for the protein encoded by Berkut allele for *TaHKT2*;*1 7AL*-*1* identified a tyrosine substitution at amino acid residue 452, located near a highly conserved domain in the 4th P-loop region which assumed a larger, neutral amino acid more hydrophobic than the negatively charged aspartic acid in the protein encoded by the Krichauff. Gene ontology for biological processes [63], Interpro Protein Domains search [64] and mutation studies [53] predicted the 4th P-loop region as a critical region in Na^+^ binding and transport and the role of wheat *HKT* genes in controlling ion transport from root to leaf blade using deletion line analysis [13], providing further evidence that the protein encoded by the Berkut allele may control Na^+^ transport within the plant affecting some yield related traits under specific but not all saline environments.

## Conclusion

Bread wheat contains two distinctly different group II *HKT* gene families, *TaHKT2*;*1* and *TaHKT2*;*2*, compared with the four individual genes in rice. Therefore, duplication of a common ancestral gene containing three exons followed by independent divergence was evident during evolution and domestication of the two species. A new member of wheat group II *HKT*, *TaHKT2*;*2* was characterized from homeologous chromosome group 7L and within a short (~3 k base pairs) physical distance from the related gene family *TaHKT2*;*1*. The *TaHKT2*;*2* multigene family consisted of three putative functional genes that were expressed in root, sheath and leaf blade in control and 200 mM NaCl stress conditions with distinct differences to *TaHKT2*;*1*. The proteins encoded by *TaHKT2*;*2* genes were similar but not identical to those encoded by *TaHKT2*;*1. TaHKT2*;*2*, like the *TaHKT2*;*1* gene family, is unlikely to be responsible for root Na^+^ uptake from external medium but potentially involved in transporting Na^+^ from root to leaf blade or in regulating K^+^ transport and may also have broader ion selectivity and transport in the plant. Moreover, the protein encoded by *TaHKT2*;*1 7AL*-*1* may be associated with yield related traits under certain saline environments but other genes in QTL intervals may also contribute to trait variation. High resolution genetic mapping and QTL analysis would provide further evidence for the role of *TaHKT2*;*1 7AL*-*1* and other genes in controlling trait variation under different saline environments.

## Methods

### Plant material

Bread wheat variety *Triticum aestivum* L. cv. Chinese Spring, six nullisomic-tetrasomic lines (N7AT7B, N7AT7D, N7BT7A, N7BT7D, N7DT7A, N7DT7B), eight deletion lines (7AL-1, 7AS-8, 7BL-2, 7BL-7, 7DL-2, 7DL-3, 7DL-5, 7DS-5) and two ditelosomic lines (Dt7BL and Dt7BS) were kindly provided by Dr John Raupp, Wheat Genetic and Genomic Resources Centre, Kansas State University, USA. The DH mapping population derived from an F1 cross between Berkut and Krichuaff consisting of 150 individuals was previously reported [33].

### Database search and identification of wheat orthologs of rice group II *HKT* genes

The group II *HKT* genes *OsHKT2*;*1*, *OsHKT2*;*2*, *OsHKT2*;*3* and *OsHKT2*;*4* and their corresponding FL-cDNA were accessed at NCBI (Genbank accession numbers AB061311, AB061313, AJ491819 and AJ491854, respectively). The rice FL-cDNA were used as query sequences in blastn and tblastx to search the wheat survey sequence database (https://urgi.versailles.inra.fr/blast/blast.php). Wheat scaffolds containing related gene sequences were identified when blast searches revealed significant hits (E = 0.0) with rice FL-cDNA sequences. Regions within each wheat scaffold having high sequence identity with FL-cDNA of rice genes were obtained and target sequences were compared with FL-cDNA of wheat group II HKTs, *TaHKT2*;*1* (Genbank accession numbers, KJ540616, U16709, KJ540618) by local alignment using the ClustalW function in GENEIOUS 6.0.3 [65].

### Primer design and PCR amplification

Multiple sequences between wheat sequences were aligned using GENEIOUS 6.0.3 [65] and sub-genome specific sequence variants were scanned manually. PCR primers were designed and positioned for 3’ mismatch [66] to obtain gene specific amplicons. NetPrimer (http://www.premierbiosoft.com/netprimer/netprlaunch/netprlaunch.html) software was used to confirm primer compatibility and annealing temperatures. Primer details are provided in Table 2.

Genomic DNA from wheat aneuploid lines was extracted using a phenol-chloroform based method [67] and 50 ng of genomic DNA was used as template for PCR amplification. PCR reactions contained 0.5 μM of each primer (Table 2), 1× PCR buffer, 1.5 mM MgCl_2_, 0.2 mM of each deoxyribonucleotides, 1 U *taq* DNA polymerase (BIOTAQ™ DNA Polymerase, Bioline, Australia) in a 20 μl reaction volume. The cycle parameters for standard PCR were 35 cycles of 94 °C 30 s, primer annealing temperature (Table 2) for 30 s, 72 °C 45 s and the final extension 7 min at 72 °C; and for touch down PCR were five cycles of 94 °C 30 s, 55–50 °C 30 s, 72 °C 45 s; and then 35 cycles of 94 °C 30 s, 50 °C 30 s, 72 °C 45 s and the final extension 7 min at 72 °C. PCR products were separated on 1 % agarose gel in 0.5× Tris-acetate EDTA at constant voltage (90 V) for 30 min and the gels were stained with ethidium bromide and visualized under UV light using Gel Doc System (BioRad, Italy). Sub-genome specificity of the PCR primers were confirmed by the absence of amplicons from gene specific PCR using nullisomic-tetrasomic lines N7AT7B, N7AT7D, N7BT7A, N7BT7D, N7DT7A, N7DT7B as template DNA.

### FL-cDNA cloning and sequencing

The development of plant material, total RNA extraction from root tissue and synthesis of cDNA were done as described below. Gene specific primers were designed to amplify multiple fragments of overlapping cDNA for the predicted coding regions of full length wheat genes (Table 2). Gene specific RT-PCR primers including 2;2AF1/2;2AR1, 2;2AF2/2;2AR2, 2;2CF1/2;2BR1, 2;2BF2/2;2BR2, 2;2BF3/2;2ADR1, 2;2ADCF1/2;2DR1, 2;2DF2/2;2ADR1 (Table 2) were used to amplify cDNA from root tissue. RT-PCR were done at annealing temperatures for each primer combination (Table 2) in 20 μl PCR reaction mixture containing 0.5 μM of each primer (Table 2), 1× PCR buffer, 1.5 mM MgCl_2_, 0.2 mM of each deoxyribonucleotides, 1 U *taq* DNA polymerase (BIOTAQ™ DNA Polymerase, Bioline, Australia). The thermocycling conditions for standard PCR were 35 cycles of 94 °C 30 s, primer annealing temperature (Table 2) for 30 s, 72 °C 45 s and the final extension 7 min at 72 °C; and for touch down PCR were five cycles of 94 °C 30 s, 55–50 °C 30 s, 72 °C 45 s; and then 35 cycles of 94 °C 30 s, 50 °C 30 s, 72 °C 45 s and the final extension 7 min at 72 °C. RT-PCR products were separated on 1 % agarose gel in 0.5× Tris-acetate EDTA at constant voltage (90 V) for 30 min and visualized under UV light using Gel Doc System (BioRad, Italy) after staining with ethidium bromide. The RT-PCR amplicons were excised from agarose gels, purified using Wizard® SV Gel and PCR Clean-Up System (Promega, CA, USA), and cloned into pGEM®-T Easy Vector Systems as per manufacturer’s recommendation (Promega, CA, USA). DNA templates from 3 recombinant bacterial colonies and representing each overlapping cDNA fragment were isolated and purified by Wizard® Plus SV Minipreps as per manufacturer’s recommendation (Promega, CA, USA) and sequenced using BigDye™ sequencing chemistry (Applied Biosystems, Perkin Elmer, Weiterstadt, Germany) using the M13 universal primers Forward primer (5’-CGCCAGGGTTTTCCCAGTCACGAC-3’) and Reverse primer 5‘-TCACACAGGAAACAGCTATGAC-3’). Sequences of overlapping cDNA for each wheat gene were assembled and aligned with the cognate genomic DNA sequence using GENEIOUS 6.0.3 [65].

### In-silico protein analysis and 3-D modelling

Proteins were deduced from FL-cDNA of the wheat genes and amino acid sequence alignments of predicted proteins were obtained using GENEIOUS 6.0.3 [65]. The predicted proteins were analysed for hydrophobicity and membrane topology using TopRED2 [68] and MPEx 3.2 [69] software using default parameters. Three dimensional structures of the proteins were predicted by PHYRE 2 [70]. The 3-D models were analysed by the PyMOL viewer and molecular Graphics system, Version 1.5.0.4 (Schrödinger, LLC).

### In-silico analysis of intergenic and promoter regions

The intergenic region between group II *HKT* genes were retrieved from rice genome browser release 7 (http://rice.plantbiology.msu.edu/) and the wheat sequence scaffolds from the International Wheat Genome Sequence (http://www.wheatgenome.org/). The NCBI nucleotide and protein databases were searched using blastn and tblastx and intergenic regions as query to identify DNA sequence identity with expressed sequence tags, partial or FL-cDNA with E-value = 0. Repetitive elements were identified using RepeatMasker (http://www.repeatmasker.org/) and EMBOSS einverted [71] software. Tandem repeat sequences were identified through self-by-self search in “Align sequences nucleotide BLAST” in the NCBI blastn suite. Transverse repeats were further analysed by blastn search function in TREP [72] and P-Mite (Plant MITE) [73] databases. C*is*-acting regulatory elements associated with salt stress response in the promoter regions were identified by blastn search in PlantCARE [74] and PLACE [75] databases.

### Hydroponic screening and development of tissue samples for expression analysis

Seeds of bread *T. aestivum* var. Chinese Spring were washed in 0.04 % sodium hypochlorite (42 g/L) for 30 s, thoroughly rinsed in deionized water before germinating on a floating wire mesh in aerated 0.1 strength nutrient solution under dark conditions. Nutrient composition at full strength was: (mM) K^+^, 3.95; Ca^2+^, 4; Mg^2+^, 0.4; NH4^+^, 0.625; NO_3_^−^, 4.375; SO_4_^2−^, 1.9; HPO_4_^2−^, 0.2; Fe-EDTA, 0.05; MES, 1.0; and micronutrients of one quarter-concentration in Hoagland solution (pH was adjusted to 6.5 using KOH). After 3 days seedlings were transferred to 0.25 strength nutrient solution in light for 1 day. Four days old seedlings were then transferred to full strength nutrient solution in aerated, foil-covered, 4.5 L pots. Seedlings were held upright by a foam holder at the stem base inserted into individual holes in the pot lids. There were four replicates per each treatment in complete randomized design. The experiment was carried out in a temperature controlled phytotron (20 ± 3 °C/15 ± 2 °C day/night) and the photosynthetically active radiation recorded at midday was at ca. 1400–1500 μmol m − 2 s − 1. Solution levels in pots were maintained by topping up with deionized water.

Ten days after initiation of the experiments and at the 2.2 Haun leaf development stage 200 mM NaCl treatments applied to designated treatment pots in 50 mM increments at 12 h intervals and the plants were maintained at the final 200 mM concentration for a full 3 days (72 h). No NaCl was added in the control pots. After 72 h samples of bulk leaf blades, sheaths and roots from each plant were sampled separately for tissue Na^+^ analysis. Prior to excision, roots were washed three times for 10 s each in a solution containing 4 mM CaSO_4_ and 368 mM mannitol (200 mM treated plants) or in 4 mM CaSO_4_ (plants subjected to no added NaCl). Leaves and sheath samples were rinsed in deionized water. All tissue samples for ion analyses were oven-dried at 70 °C, weighed and then ground to a powder. Samples of dried powdered tissue (100 mg) were extracted in 5 ml of 0.5 M HNO_3_ for 3 days on a mechanical shaker in dark conditions. Tissue Na^+^ concentrations were measured in technical triplicate using a flame photometer (Sherwood 410, Cambridge) and mean values represented each treatment replicate tissue sample. The reliability of the methods was confirmed by analyses of a reference tissue (broccoli, ASPAC Plant number 85) taken through the same procedures.

### RNA extraction and RT-PCR

Development of unstressed and salt treated seedlings of wheat *T. aestivum* var. Chinese Spring was done as described above. Bulk leaf blade, sheath and roots were harvested from control and treated plants at the end of 72 h treatment period, snap frozen in liquid N_2_ for total RNA extraction. Total RNA was extracted from frozen tissues using a modified protocol by combining the TRIzol protocol, (Invitrogen, Carlsbad, CA) and ISOLATE II RNA Plant Kit (Bioline USA Inc, Taunton). Approximately 100 mg of tissue sample was grinded in liquid N_2_ and 1 mL of TRIzol reagent was added per 100 g of grinded tissue. Samples were incubated in TRIzol for 5 min at room temperature and extracted with 0.2 ml chloroform per 1 ml Trizol shaking vigorously by hand for 15–30 s. The samples were incubated at room temperature for 2 min and centrifuged at 12,000 g for 15 min at 4 °C. The aqueous phase was transferred to a clean RNase free tube avoiding the interphase and RNA was precipitated by adding 0.5 ml of isopropanol and incubating 10 min at room temperature. The isoproponal mixture was loaded on a ISOLATE II RNA Plant Column from the ISOLATE II RNA Plant Kit (Bioline USA Inc, Taunton) and the extraction was continued using the manufacturers’ recommendation. RNA purity and the quantity were determined by UV spectrophotometry at 260 and 280 nm (A^260^/A^280^ ~ 1.9 and A^260^/A^230^ > 2). First strand cDNA was synthesised using SensiFAST™ cDNA Synthesis Kit (Bioline USA Inc, Taunton) using 1 μg of total RNA in a 20 μl reaction following instructions by the manufacturer. Integrity of synthesised cDNA was verified by RT-PCR using primers designed for glyceraldehyde-3-phosphate dehydrogenase (GAPDH) gene (Table 2).

### Quantitative real time PCR (qRT-PCR)

The qRT-PCR reactions was done in a Rotor-Gene™ 3000 (Corbett Research Ltd, UK) using SensiMix SYBR® No-ROX mix (Bioline USA Inc, Taunton) master mix. Three independent biological replicates of leaf, root and sheath tissues were used for analysis of transcript abundance of each gene under control and 200 mM NaCl treated conditions. Three technical replicates were used for each biological sample. The qRT-PCR reaction consisted of a final volume of 10 μl containing 5 μl of SensiMix SYBR® No-ROX mix (Bioline USA Inc, Taunton), 0.4 μM of each primer (Table 2) and 3 μl of a 1 in 20 dilution of synthesized cDNA and analysed using the following conditions: 3 min at 94 °C, 40 cycles at 94 °C for 15 s, 60 °C for 10s, and 72 °C for 20s. SYBR signals were acquired at the completion of the primer annealing step. Integrity and specificity of qRT-PCR was confirmed by melting curve analysis (55–95 °C with a heating rate of 0.5 °C/min) and agarose gel electrophoresis of the amplicons. The cDNA samples served as a template for stability analyses using three internal reference genes *TaEFA*, GAPDH and *TaActin* using similar qRT-PCR reaction conditions. The C_T_ values obtained for each gene using control and 200 mM NaCl treated leaf, root and sheath cDNA were subjected to analysis of variance and genes showing stable expression in samples were selected as internal references for expression normalization of *TaHKT2* genes. Primer sequences for the six genes of interest and reference genes are listed in Table 2. The relative change of transcript abundance in NaCl treated samples were estimated by 2^-∆∆CT^ method [76]. Standard error in 2^-∆∆C^ was estimated by predicting RQ_Min_ and RQ_Max_ [77, 78].

### Deletion bin mapping

Wheat genes were bin mapped using eight deletion lines 7AL-1, 7AS-8, 7BL-2, 7BL-7, 7DL-2, 7DL-3, 7DL-5 and 7DS-5 and ditelosomic lines Dt7BL and Dt7BS. Genomic DNA from deletion and ditelosomic lines were extracted following a phenol-chloroform based method previously described [67] and used as template for PCR amplification. The location of genes within each deletion bin was determined by the presence or absence of amplicons from corresponding deletion or ditelosomic lines for gene specific PCR using the primer pairs 2;2AF1/2;2AR1, 2;2CF1/2;2BR1 and 2;2ADCF1/2;2DR1 (Table 2). The PCR reaction conditions were same as described above. PCR products were analysed on 1 % agarose gel in 0.5× Tris-acetate EDTA (constant voltage (90 V) for 30 min) and visualized under UV light using Gel Doc System (BioRad, Italy) after staining with ethidium bromide.

The chromosomal bin location of the flanking markers for the Na^+^ exclusion QTLs and other QTLs associated with salt related traits reported in the Berkut/ Krichauff DH population [33] were obtained from deletion bin mapping in [79, 80]. The bin location of the *wmc139* SSR marker was experimentally determined by PCR using deletion lines as template DNA and primers 5’-TGTAACTGAGGGCCATGAAT-3’ and 5’-CATCGACTCACAACTAGGGT-3’ obtained from the GrainGene database (http://wheat.pw.usda.gov/GG3/). Touch down PCR was performed at 65–55 °C as described above.

### Cleaved amplified polymorphic sequence (CAPS) marker development

*TaHKT2*;*2 7AL*-*1* genes were amplified, cloned and sequenced from Berkut and Krichauff using gene specific primers as previously described [13]. Similarly *TaHKT2*;*2 7AL*-*1* gene was cloned and sequenced from both parents using the primers 2;2AF1/2;2AR1, 2;2AF2/2;2AR2, 2;2AF3/2;2ADR1 as described above. Gene sequences were searched for DNA polymorphism by multiple sequence alignments using GENEIOUS 6.0.3 [65]. A gene specific CAPS marker polymorphic for Berkut and Krichauff parents was developed for *TaHKT2*;*1 7AL*-*1* by amplifying a 1103 base pairs fragment using primers 2;1AF1 and 2;1AR1 (Table 2) in PCR reactions and thermocylcing conditions as described above. PCR products (10 μl) were digested with 10 U of *Xmn*1 (Promega, CA, USA) by incubation at 37 °C for 2 h in a reaction mixture as per the manufacturers recommendation (Promega, CA, USA). The digested products were separated on 1.5 % agarose at 0.5× Tris-acetate EDTA (constant voltage of 90 V for 30 min) and visualized under UV light using Gel Doc System (BioRad, Italy) after staining with ethidium bromide.

### Genetic mapping of group II *HKT*s

A total of 150 DH lines from the Berkut/Krichauff mapping population were grown in greenhouse and genomic DNA was extracted from leaf material using a phenol-chloroform based method described in [67], quantified by Nanodrop_ND_1000 v3.2 spectrometer (Thermo Fisher Inc., DE, USA) and DNA concentrations adjusted to 50 ng/μl. The 150 DH lines were genotyped for *TaHKT2*;*1 7AL*-*1* gene specific CAPS marker and allelic data integrated into existing genetic maps of simple sequence repeat (SSR) and Diversity Array Technology (DArT) markers described in [33]. Marker allele data for *TaHKT2*;*1 7AL*-*1* was assigned in the genetic map using the “Distribute” command,, linkage criteria = 0.01, map function = Kosambi followed by the “Ripple” command in MapManagerQTX version QTXb20 [81]. The chromosome linkage and QTL intervals were graphically presented using MapChart V2.2 [82].

### Availability of supporting data

The data supporting the results of this article are included within the article and its additional files.

## Additional files

Additional file 1: Figure S1. Amino acid sequence alignments of TaHKT2;1 and TaHKT2;2 proteins. Glycine molecules composing the cation selectivity filter domains are indicated by a red line. (PPTX 758 kb) Additional file 2: Figure S2. DNA Sequence alignment of the *OsHKT2*;*1*-*OsHKT2*;*4* intergenic region (A) and *TaHKT2*;*1*-*TaHKT2*;*2* intergenic region on *7AL*, *7BL* and *7DL* (B). The complete sequence of PIF/Harbinger type MITE is highlighted in purple, remnants of a CACTA type transposon “DTC_Isidor” in blue and individual motifs of tandem duplications are in red, yellow, green and dark blue. Boxed sequences represent salt activated *cis*-acting regulatory elements and other regulatory motifs in the *OsHKT2*;*4* and *TaHKT2*;*2* promoter regions. (RTF 545 kb)

## Abbreviations

CAPS: cleaved amplified polymorphic sequence
CRE: *cis*-acting regulatory elements
DArT: diversity array technology
DH: doubled haploid
FL-cDNA: full length cDNA
GAPDH: glyceraldehyde-3-phosphate dehydrogenase
HKT: high affinity potassium transporter
INDEL: insertion-deletion
MITE: miniature inverted transposon element
NaCl: sodium chloride
NT: nullisomic-tetrasomic
qRT-PCR: quantitative reverse transcription polymerase chain reaction
QTL: quantitative trait loci
RT-PCR: reverse transcription polymerase chain reaction
SNP: single nucleotide polymorphism
SOS: salt overly sensitive
SSR: simple sequence repeat
TF: transcription factor
