# Supplementary figures and images for: A comparative gene analysis with rice identified orthologous group II HKT genes and their association with Na+ concentration in bread wheat
Source: BMC Plant Biol. 2016 Jan 19;16:21. doi: 10.1186/s12870-016-0714-7 (PMC4719669; doi:10.1186/s12870-016-0714-7)

## Slide 1
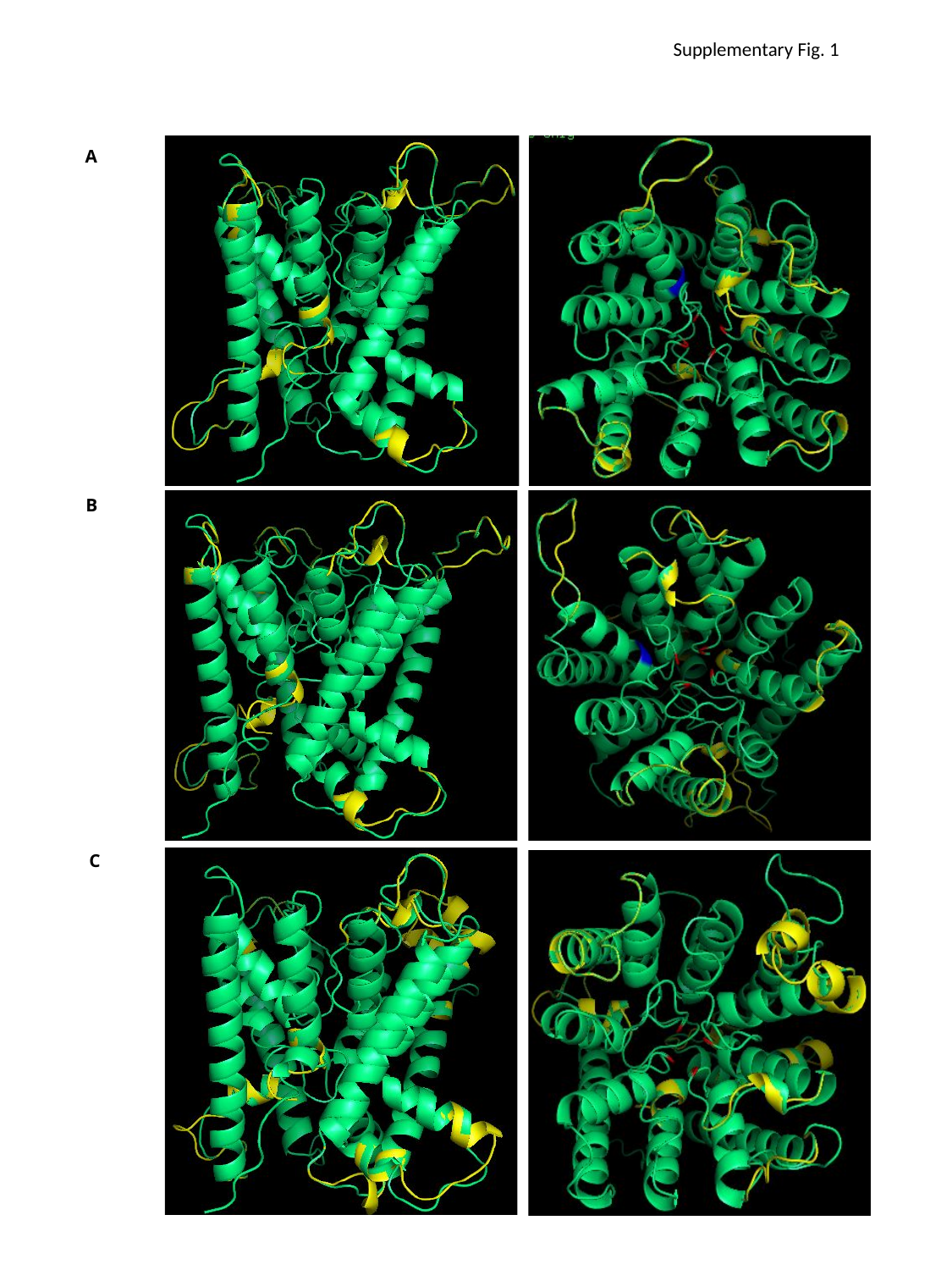

Supplementary Fig. 1
A
B
C

Supplement: Additional file 1: Figure S1. — Amino acid sequence alignments of TaHKT2;1 and TaHKT2;2 proteins. Glycine molecules composing the cation selectivity filter domains are indicated by a red line. (PPTX 758 kb) [file 12870_2016_714_MOESM1_ESM.pptx]
