# Supplementary material for: A comparative gene analysis with rice identified orthologous group II HKT genes and their association with Na+ concentration in bread wheat
Source: BMC Plant Biol. 2016 Jan 19;16:21. doi: 10.1186/s12870-016-0714-7 (PMC4719669; doi:10.1186/s12870-016-0714-7)
Supplement: Additional file 2: Figure S2. — DNA Sequence alignment of the OsHKT2;1-OsHKT2;4 intergenic region (A) and TaHKT2;1-TaHKT2;2 intergenic region on 7AL, 7BL and 7DL (B). The complete sequence of PIF/Harbinger type MITE is highlighted in purple, remnants of a CACTA type transposon “DTC_Isidor” in blue and individual motifs of tandem duplications are in red, yellow, green and dark blue. Boxed sequences represent salt activated cis-acting regulatory elements and other regulatory motifs in the OsHKT2;4 and TaHKT2;2 promoter regions. (RTF 545 kb) [file 12870_2016_714_MOESM2_ESM.rtf]

 


        10         20         30         40         50         60         70         80         90        100        110        120        130        140        150                                     
....|....| ....|....| ....|....| ....|....| ....|....| ....|....| ....|....| ....|....| ....|....| ....|....| ....|....| ....|....| ....|....| ....|....| ....|....| 
---------- -MPVRLHTFL SSARHISNSS VFIFQFIAFH LSPLLIHLSY FVVIDVLGFA ALMALKPSNP NYSPRYVDIF FLSTSAVTVT GLATIKMEDL STSQVVILTL LMLLGSEMFV SLLGHIHELS KQNKHDPEDS RVTSVTVQ--  
---------- -MPIRLPIFL SSARHVSNSS VFIFRFIAFH LSPLLIHLSY FVIIDVLGFV ALMALKPSNP NYSPRYVDIF FLSTSAVTVT GLATIKMEDL SSSQVVVLTL LMLLGSEMFV SLLGHIHELS KQNKHDPEDS RVRSVTVQ--  
---------- -MPIRLHTFL SSARHVSNSS VFIFQFIAFH LSPLLIHLSY FVIVDVLGFA ALMALKPSNP NYSPRYVDIF FLSTSAVTVT GLATIKMEDL STSQVVILTL LMLLGSEMFV SLLGHIHELS KQNKHDPEDS RVRSVTVQ--  
MGRVKRFYQD FIHIKLHSFC RISRYVVDSI AFVYRFVALH VHPFWIQLSY FLAIAILGSV LLMSLKPSNP DFSPPYIDML FLSTSALTVS GLSTITMEDL SSSQIVVLTL LMLIGGEIFV SLLGLMLRVN HQDMQDLPSV KISSVPVELE  
MDRVKRFYQD FIHIKLHSFS RISRYVVDSI VFIYRFVALH VHPFWIQLSY FLAIAILGSV LLISLKPSNP EFSPPYIDML YLSTSALTVS GLSTVKMEDL SSSQIVVLTL LMLVGGEIFV SLLGLMLRVN HQDMQDLPSV KISSVPVELE  
MGRVKRFYQD FIHIKLHSFS RISRYVVDSM VFIYRFVALH VHPFWIQLSY FLAMAILGSV LLMSLKPSNP EFCPPYIDML YLSTSALTVS GLSTVKMEDL SSSQIVVLTL LMLVGGEIFV SLLGLMLRVN HQDMQDLPSV KISSVPVELE  


        160        170        180        190        200        210        220        230        240        250        260        270        280        290        300                            
....|....| ....|....| ....|....| ....|....| ....|....| ....|....| ....|....| ....|....| ....|....| ....|....| ....|....| ....|....| ....|....| ....|....| ....|....| 
---------- -DESQIEEAI PATPSINTTS LKKS-CRKYI GFVLLAYMVL ILLVGSLLVF LYVAHVSTAR DVLTRKSINT MLFSISVTVS SFTNGGLIPT NESMAVFSSN QGLLLLLTGQ ILAGNTLLPV FLRLMIWALR GLRITRAKPE  
---------- -DESQIEEAI PATPSINTIS LKKS-CLRYI GFVLLAYMVS ILLVGSLLVF LYVAHVSTAR DVLTRKSINT MLFSISVTVS SFTNGGLIPT NESMAVFSSN QGLLLLLTGQ ILAGNTLLPV FLRLVIWALR GLRITRAKPE  
---------- -DESQIEEAI PVTPSTNTTS LKKG-CRKYI GFVLLAYMVL ILLVGSLLVF LYVAHVSTAR DVLTRKSINT MLFSISITVS SFTNGGLIPT NESMAVFSSN QGLLLLLTGQ ILAGNTLLPV FLRLVIWALR GLSITRAKPE  
ELDLPNSMAL CDESQLEEAA HAIPPKKCTE LKRSRSVKCL GYVVFGYFAM IHVLGFLLVF LYITHVPTAS APLNKKGINI VLFSLSVTVA SCANAGLVPT NENMVIFSKN SGLLLLLSGQ MLAGNTLFPL FLRLLVWFLG --RITKVK--  
VLDLANSMAL CDESQLEDAS HAIPPKKCTE LKRSRSVKCL GYVVFGYFAV IHVLGFVLVF LYITHVPTAS APLNKKGINI VLFSLSVTVA SCANAGLVPT NENMVIFSKN SGLLLLLSGQ MLAGNTLFPL FLRLLVWFLG --KLTKVK--  
VLDLANSMAL CDESQLEEAA HAIPPKKCTE LKRTESVKCF GHVIFGYFAV IHVLGFLLVF LYITHVPTAS APLNKKGINI VLFSLSVTVS SFANAGLVPT NENMVIFSKN SGLLLLLSGQ MLAGNTLFPL FLRLLVWFLG --KLTKVK--  


        310        320        330        340        350        360        370        380        390        400        410        420        430        440        450                            
....|....| ....|....| ....|....| ....|....| ....|....| ....|....| ....|....| ....|....| ....|....| ....|....| ....|....| ....|....| ....|....| ....|....| ....|....| 
ELEFMMNNTK ALGSNHLLPN KQTVFLAASV AALIAVAVTF FCCLNWESAV FAGLTPNQKI TNALFMAVNT RQAGENSIDC SLVAPAVLIL FIAMMCIPAS TSFLSLHEGA ERGITEHKDG ANKRRMSLNK MLFSPLACTA VLIMLACITE  
EFEFMMNNTK GVGFNHLLPT QQTVFLTASV AALIAVAITL LCCLNWNSAV FAGLTPNQKI TNALFMAVNT RQAGENSIDC SLVAPAALVL FIAMWCIPAS TSFLSLHERD KRGITEHKDG ANKRRLSLNK MLFSPLACTA VLIMLVCITE  
ELEFMMNNTK ALGSNHLLPN KQTVFLAASV AALIAVAVTF FCCLNWESAV FAGLTPNQKI TNALFMAVNT RQAGENSIDC SLVAPAVLIL FIAMMCIPAS TSFLSLHEHA ERGITEHKDG ANKRRLSLNK MLFSPLACTA VLIMLVCITE  
ELRLMINNPE EVRFANLLAR LPTVFLSSTV VGLVAAGVTM FCAVDWNSSV FDGLSSYQKT VNAFFMVVNA RHSGENSIDC SLMSPAIIVL FIVMMYLPSS ATF-APPSGD TKTTNENTKG KVKRGSLVQN LAFSPLGCNI IFVMVACITE  
ELRLMTKNPE EVHFANLLPR LPTVFLSSTV IGIVAAGVTL FCSVDWNSSV FDGLGSYQKT VNAFFMVVNA RHSGENSIDC SLMSPAIVVL FIGMMYLPSS ATF-APPSGD TKTTNENTKG KGKRGSLVQN LAFSPLGCNI IFVIVACITE  
ELRLMIKNPE EVHFANLLPG LRTVFLSSTV VGLAAAGVTL FCAVDWNSSV FDGLSSYQKT VNAFFMVVNA RHSGENSIDC SLMSPAIIVL FIIMMYLPSS ATF-APPNGD TKTTDASTKG --KRGSLVQN LVFSPLGCNI IFVIVACITE  


        460        470        480        490        500        510        520        530                      
....|....| ....|....| ....|....| ....|....| ....|....| ....|....| ....|....| ....|....| ....|...
RRSLSADPHN FSTFNMIFEV ISAYRNVGLS TGYSCARLPH AEKQSVCQDM PYSFSGWWSD QGKVVLVLVM LYGRLKCFHR QRS----- 
RRSLSADPLN FSTFNMIFEV ISAYRNVGLS IGYSCARLPH PEKQSVCQDM PYSFSGWWSD QGKVVLVLVM LCGRLKCFHR QRS----- 
RRSLSADPLN FSTFNMIFEV ISAYRNVGLS TGYSCARLPH PEKQSVCQDM PYSFSGWWSD QGKVVLVLVM LCGRLKCFHR QRS----- 
RRRLRNDPLN FSTLNMIFEV ISAYGNAGLS TGYSCSRLHQ LHPEIICQDK PYSFSGWWSD GGKFVLILVM LYGRLKAFTL ATGKSWKV 
RRRLRSDPLN FSTLNMIFEV ISAYGNVGLS TGYSCSRLHQ LHPEIICQDM PYSFSGWWSD GGKFLLVLVM LYGRLKVFAV STGKSWKV 
RRRLRNDPLN FSTLNMIFEV ISAYGNVGLS TGYSCSRLHQ LHPEIICQDK PYSFSGWWSD GGKFLLVLVM LYGRLKAFAV STGKSWKV 
